# SistematX, an Online Web-Based Cheminformatics Tool for Data Management of Secondary Metabolites

**DOI:** 10.3390/molecules23010103

**Published:** 2018-01-03

**Authors:** Marcus Tullius Scotti, Chonny Herrera-Acevedo, Tiago Branquinho Oliveira, Renan Paiva Oliveira Costa, Silas Yudi Konno de Oliveira Santos, Ricardo Pereira Rodrigues, Luciana Scotti, Fernando Batista Da-Costa

**Affiliations:** 1 Postgraduate Program in Natural Products and Synthetic Bioactive, IPeFarM, Federal University of Paraíba, Campus I, Cidade Universitária, João Pessoa 58051-900, PB, Brazil; chonny622@gmail.com (C.H.-A.); renan0paiva@hotmail.com (R.P.O.C.); syudik12@gmail.com (S.Y.K.d.O.S.); ricardo.p.rodrigues@ufes.br (R.P.R.); luciana.scotti@gmail.com (L.S.); 2 Department of Pharmacy, Federal University of Sergipe (UFS-SE), Av. Marechal Rondon s/n, Jd. Rosa Elze, São Cristóvão 49100-000, SE, Brazil; tiago.branquinho@ufs.br; 3 AsterBioChem Research Team, Laboratory of Pharmacognosy, School of Pharmaceutical Sciences of Ribeirão Preto, University of São Paulo (USP), Av. do Café s/n, Ribeirão Preto 14040-903, SP, Brazil; febcosta@fcfrp.usp.br

**Keywords:** SistematX, secondary metabolites, data management, online web-based tool

## Abstract

The traditional work of a natural products researcher consists in large part of time-consuming experimental work, collecting biota to prepare and analyze extracts and to identify innovative metabolites. However, along this long scientific path, much information is lost or restricted to a specific niche. The large amounts of data already produced and the science of metabolomics reveal new questions: Are these compounds known or new? How fast can this information be obtained? To answer these and other relevant questions, an appropriate procedure to correctly store information on the data retrieved from the discovered metabolites is necessary. The SistematX (http://sistematx.ufpb.br) interface is implemented considering the following aspects: (a) the ability to search by structure, SMILES (Simplified Molecular-Input Line-Entry System) code, compound name and species; (b) the ability to save chemical structures found by searching; (c) compound data results include important characteristics for natural products chemistry; and (d) the user can find specific information for taxonomic rank (from family to species) of the plant from which the compound was isolated, the searched-for molecule, and the bibliographic reference and Global Positioning System (GPS) coordinates. The SistematX homepage allows the user to log into the data management area using a login name and password and gain access to administration pages. In this article, we introduced a modern and innovative web interface for the management of a secondary metabolite database. With its multiplatform design, it is able to be properly consulted via the internet and managed from any accredited computer. The interface provided by SistematX contains a wealth of useful information for the scientific community about natural products, highlighting the locations of species from which compounds are isolated.

## 1. Introduction

The traditional work of a natural products researcher can be summarized as collection of biological samples, preparation of extracts for biological screening or bioassay-guided fractionation, and isolation and purification of (bioactive or not) compounds. However, the first question that may arise is the following: are these compounds known or new? In addition, metabolomics studies have introduced a new question: how fast can this information be obtained [1]?

The stage of dereplication, a process known as the rapid characterization of previously known compounds in mixtures without their prior purification, has become a strategically important area for natural products research involved in screening programs in several commercial and non-commercial databases [2,3,4]. These databases can be searched with minimal information, such as structural chemical and biological data from compounds; however, dereplication now requires additional information, such as biogeographical and taxonomic information, or the presence of a certain compound (new or known) in other individuals of the same species, genus, subfamily, and family. This information can also help to reduce the number of hits during chemical identification by dereplication.

Large structure-based data collections, such as ChemSpider, PubChem, ChemBl, and ZINC [5,6,7,8] can be used for this purpose [9,10]. However, these databases are not specialized in secondary metabolite information that is valuable to the natural products researchers, for example, botanical occurrence and geographical localization. For this reason, a number of specialized natural products databases were developed that are commercially or freely available and only contain restricted information, for example, the Dictionary of Natural Products (DNP) [11], NAPRALERT [12], Marinlit for marine natural products [13], and Antibase for microorganisms and higher fungi materials. Nevertheless, none of these provide structural collections in a format that can be rapidly integrated into software such as ACD/Structure Elucidator and others [9].

Other natural products databases provide natural products extracted from various resources and contain various associated information such as toxicity prediction, but so far, little or nothing is known about these resources, for example, SUPER NATURAL II [14]. Natural products databases exhibit a huge range of structural complexity and thus are expected to contribute to the ability of such databases to provide positive hits [2,15]. These structures are available in regional databases, for example, NUBBEdb [16], SANCDB [17], TM-CM [18], TCM-Database@Taiwan [19], NANPDB [20], and TCMID [21]. Many have been used in virtual screening research studies. In addition to the database information described above that uses two-dimensional (2D) structures, several databases have selected methods and tools for generating three-dimensional (3D) structures of small organic molecules, often for use in structure-based drug design.

In addition, databases of natural products with a focus on metabolomic studies with relationships between species-metabolites include the KNApSAcK Family [22], TIPdb-3D [23], and AsterDB [24], which enable searches for chemical structures by plant species names and other taxonomic information. Nevertheless, some data are still lacking for the purpose of exact dereplication. Information such as exact mass and geographic data can be very important for this type of study [25,26,27].

It is not enough simply to focus on the information contained in a database. A clean and user-friendly interface, fast search, and consistency between currently available operating systems (Microsoft Windows, Mac, and Linux) can be just as important. For this purpose, the SistematX software was developed to provide the abovementioned information for chemosystematics studies, dereplication, and botanical correlations.

## 2. Results and Discussion

### 2.1. Utility and Discussion

**Figure 1:**
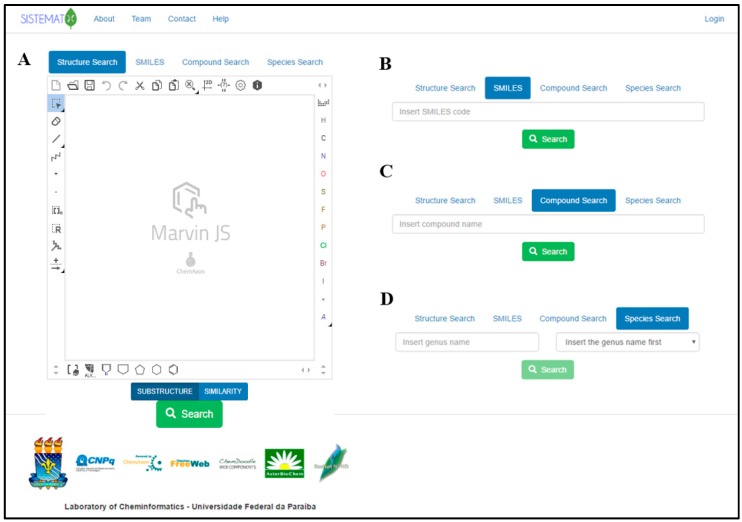
SistematX homepage with different search options: (**A**) by structure; (**B**) by Simplified Molecular-Input Line-Entry System (SMILES); (**C**) by compound name; and (**D**) by plant species.

The SistematX homepage is shown in Figure 1A. After the user enters the website (http://sistematx.ufpb.br), the “Structure search” option is seen with the MarvinJS API (Application Programming Interface) at the top of the screen. Another three search options can be exhibited in the interface. The initial screen of the system also shows the SMILES (Simplified Molecular-Input Line-Entry System) code (Figure 1B), compound name (Figure 1C) and plant species search modes (Figure 1D).

In the first option, the user can perform the search using the drawn full structure or molecular skeleton, fragments, or substructures, which is important in cases when the user only knows a structural characteristic of the structure such as functional groups or when studies require structural similarity, structural groups or families of compounds. It is possible to use the similarity search option that is currently available in SistematX. The search results page shows all compounds that correspond to the value above the cut of provided by the user in the decreasing order of similarity, showing the similarity values on the top. A substructure and similarity search is performed using a hashed fingerprint. Special molecular features are present in the query (e.g., stereochemistry, charge), only those targets match that also contain the feature. However, if a feature is missing from the query, it is not required to be missing.

In addition, it is possible to search by SMILES code, a chemical notation system capable of representing even the most complex organic compounds using a simple grammar that is very well known to organic chemistry researchers; for this reason we add this option separately from the structure search using the MarvinJS API, being friendly for one just to copy and paste the SMILES code (Figure 1B); by common (usual) name or IUPAC (International Union of Pure and Applied Chemistry) name (or part of one of these); and by species, although in this option, it is necessary to first insert the name of the genus (which presents an autocompletion option). After being selected, the system presents all species available for the user to select for the search.

**Figure 2:**
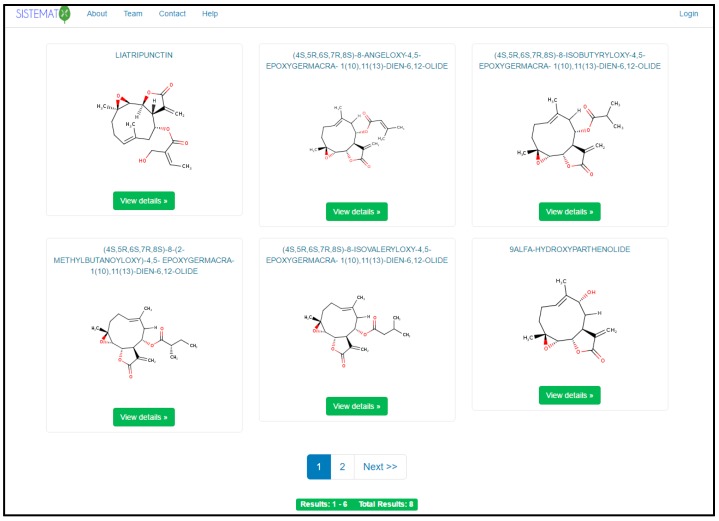
SistematX results page.

**Figure 3:**
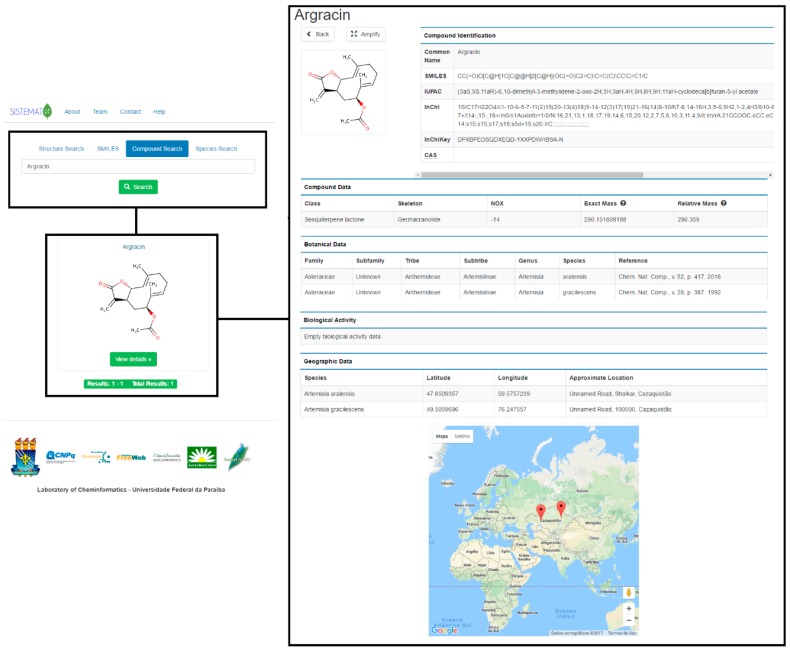
SistematX screen for molecular data.

When performing a search, the mechanism generates a search results page (six results per page), using common names; if the compound does not have one, it shows the IUPAC name (Figure 2). The user can set the number of structure results per page. When a result is selected, the user has access to the data for that molecule, which are classified into six different groups (Figure 3).

The first group of results that appears is related to the structural representation of the searched molecule. The 2D structure is observed in the interface; on top, this appears as the option to amplify. After this is clicked, the system displays the visualization of the molecule in 2D and 3D (ChemDoodle, iChemLabs, Piscataway, NJ, USA) and an additional option for saving the 2D or 3D structure in an MDL (Molecular Design Limited, San Ramon, CA, USA) Molfile. The second type of result exhibited by the systems associated with compound identification, such as common name, SMILES code, IUPAC name, InChI (IUPAC International Chemical Identifier, Research Triangle Park, NC, USA) code, InChIKey code and CAS (Chemical Abstracts Service, Columbus, OH, USA) number. Except for the common name, which is optional and registered by the administrator, all parameters are provided by the JChem API.

Compound data results include important characteristics for natural products chemistry. The class of secondary metabolite of the searched molecule and its skeleton provide information about its biosynthetic pathway and assists in chemosystematics and chemotaxonomic studies. Oxidation number (NOX), which is calculated based on the Hendrickson rules [28], has been fundamental in chemotaxonomy since Gottlieb related the oxidation grade of molecules to species evolution [29]. Molecular mass is calculated using the most abundant isotope of each element (exact mass) and the average atomic mass of each element (relative mass); these data are important for users working on purification processes and for structural elucidation of molecules, due to the mass information, which is essential for determining the purity of secondary metabolites.

In the botanical data field, the user can find specific information such as the taxonomic rank (from family to species) of the plant from which the compound was isolated, the searched molecule, and the bibliographic reference, which includes journal name, volume, page and year. Because many different species can biosynthesize the same molecule, there is one register per species. Meanwhile, the biological data exhibit results obtained in studies related to the biological activity of the searched molecule; the type of activity, system, units, activity value and bibliographic reference are available in this section.

Plant species have revealed clear genetic signals for local adaptation [30]. One species can synthesize a secondary metabolite depending on its location, and there are observed variations in compound concentrations at different sites. Because geographical data is an important parameter in natural products research, SistematX shows geographical coordinates (latitude and longitude) for a searched molecule and an approximate location of the species from which this metabolite was isolated. Using the Google Maps API, the user can observe the species location on the world map.

### 2.2. Data Management

**Figure 4:**
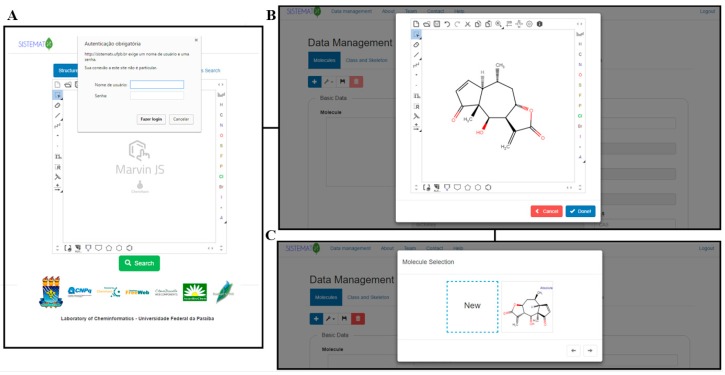
SistematX creates new registers through an administrator: (**A**) login and password option; (**B**) structure view and (**C**) molecule selection.

On the SistematX homepage, the user can also log into the data management area using login name and password (Figure 4A) and from there access the administration pages to edit or register new molecules. Once the corresponding information has been accepted, the data management interface appears.

**Figure 5:**
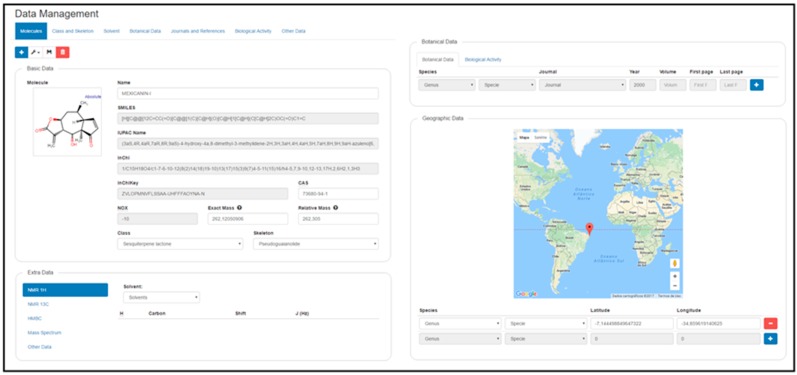
SistematX data management interface.

The first requirement to register a new molecule is to insert the structure in the MarvinJS API (Figure 4B). Several methods can be used to accomplish this step: drawing, copying SMILES code, or importing the molecule in a compatible format (e.g., sdf, cdx, mol, mol2). The molecule selection option then appears (Figure 4C), and if the molecule is new to the system, it appears with the option New. If this option is selected, a blank register page with four subdivisions is shown: Basic Data, Extra Data, Botanical Data, and Geographical Data. However, if the molecule already exists in the system, another box with the drawn molecule appears, and after choosing this box, the register page for the structure containing all previously registered information appears (Figure 5).

Immediately after the New option is selected, it appears in the register page, including some basic data generated by the MarvinJS API: SMILES, IUPAC name, InChI, InChIKey, NOX, exact mass and relative mass. The class and skeleton of metabolites must be chosen to register a new structure in the system; this is a sequential process, and thus, the class must first be selected to make the skeleton structure option available. Classes and skeletons not already registered in the system can be registered via the Class and Skeleton tab on the top of the screen. Common name and CAS are optional registration options.

The Extra Data subdivision allows insertion of all structural spectroscopic information, such as ^1^H- and ^13^C-NMR (Nuclear Magnetic Resonance) and mass spectra. In addition, it is possible to find 2D NMR information through the HMBC (Heteronuclear Multiple Bond Correlation) technique to establish the relationship between ^13^C- and ^1^H-shifts. In the NMR data, the administrator must first select the deuterated solvent used in the spectroscopic studies. If this information does not exist in the options, it can be registered by selecting the Solvent tab on the top of the screen. After the structure appears with an atomic numeration assigned by the MarvinJS API (identified as Atom), it is always necessary to verify these numbers for the biogenetic numeration (identified as “Biogenetic”) and finally to add the chemical shift value for each atom. For ^1^H-NMR, it is also possible to register H–H coupling constants (J in Hz). Mass spectrum information, molecular mass and intensity of fragments can also be being registered.

To create a new registry for botanical data of a certain species, the following information is required: journal, year, volume, first page and last page information. Journal, genus and species are drop-down lists, and these last two must be filled in this order. If any information relevant to these three fields does not exist, it can be inserted by clicking in Journal and References and writing the journal name (autocomplete tab) or by clicking in Botanical Data, where taxonomic data are registered. Biological activity data appear as activity, system, system type, value, journal, year and pages. Drop-down lists can be filled with previously registered data or by entering new data in the Biological Activity tab.

Finally, the administrator can register geographical data using the Google maps API. For any structure, it is possible to register latitude and longitude of the corresponding species studied. The genus and species boxes are filled in the same manner described above. Longitude and latitude can be inserted in two ways, first by writing the coordinates in the spaces; once registered, they appear on the world map as a red indicator showing the location. Another method is to select the place by clicking on the red pin on the map; when the pin is released, the values of longitude and latitude appear in the respective boxes.

Currently, our database has more than 1300 sesquiterpene lactones and 850 flavonoids and chalcones with more than 4000 botanical occurrences of the Asteraceae family and approximately 500 alkaloids which represents more than 750 botanical occurrences of the Apocynaceae family and several terpenes and alkaloids of Annonaceae, Apocynaceae and Asteraceae that correspond to more than 800 botanical occurrences.

## 3. Materials and Methods

### Implementation

SistematX was developed in the Java programming language version 8 or higher, using JSP (JavaServer Pages) technology version 2.1 or higher and MySQL database version 5.5.46-0 for Linux [31] to maintain the system data. An initial version of SistematX web was published in the proceedings of MOL2NET in 2015 to demonstrate its functionalities to the academic community and, with feedback, to improve old functionalities and add new tools [32]. In the current version, several functionalities were added. For example, automatically for each compound its relative mass, exact mass, CAS number, InChI (in the previous version only the InChIKey was available). The CAS number if not generate automatically, can be added manually and geographical localization of species where the compound was isolated is now available. It is possible to perform a structural search by similarity index.

The system uses JSP to create pages with specific information for each molecule and dynamic page change by clicking on certain buttons. Intermediary pages are used to recover information from the database and insert it in the JSP. The system creates a DAO (Data Access Object) to organize the data on intermediary pages, working like a bridge from the DAO to JSP. For each database table needed in a request, a DAO is created specifically for that table containing all attributes from the database table.

Bootstrap version 3.3.5 [33], a graphical interface web framework that uses HTML (HyperText Markup Language), CSS (Cascading Style Sheets) and JavaScript, is employed for a better appearance on HTML pages, adapting some functions and styles to aid in the website design. The system also uses jQuery framework version 2.1.4 [34], a powerful JavaScript tool to manipulate HTML DOM (Document Object Model) events. Another framework, jQuery AutoComplete version 1.2.18 [35], is used to generate autocomplete inputs.

Several APIs are used in the SistematX implementation (Table 1). MarvinJS version 15.7.20, from ChemAxon [36], is the drawing API that is integrated with ChemAxon JChem WebService [37], an external online service that transforms the drawn structure into SMILES code, after which a JChem API function turns it into a binary fingerprint. This fingerprint is used to search for molecules, using substructure or similarity, in the database via their structure. The molecule converted to the fingerprint is used as a fragment in the search, comparing it to the database molecule’s fingerprints to determine if it exists inside as a fragment. This API utilizes HTML, CSS, and JavaScript to perform the transformations. We use the standardizer API of Chemaxon JChem Web Services to standardize not only nitro groups or aromatize the structure search results correctly as possible, making a query and the database with similar representation.

MarvinJS is used to create a 3D structure from a 2D molecule, and ChemDoodle Web Components [38] shows this view as JavaScript in the browser. This API is able to generate several 2D and 3D molecule graphical views using pure JavaScript.

The ChemAxon API allows the visualization of compound characteristics. SistematX displays general nomenclature information such as the common name, SMILES code, IUPAC name, InChI, InChIKey, CAS registry number and properties such as oxidation number (NOX), exact mass and relative mass.

In addition, Google Maps, from Google Inc. [39], an API used to prepare maps and locations, is used in the system to show the registered metabolite location on the world map. The API draws the map and receives locations from the database, which are two variables representing the latitude and longitude. A registered molecule may have multiple locations and a species linked to it. Using a JavaScript function, it graphically sets the locations on the map. When registering by clicking on the map, it sets a marker at the mouse location and adds a line to the coordinates list below the map for each marker on the map, allowing it automatically to change the position when the value in the latitude or longitude boxes is changed. The coordinates are also transformed into an approximate address, using reverse geocode, a function from the Google Maps API.

## 4. Conclusions

In this article, we introduce a web interface for managing a secondary metabolite database, which is multiplatform and able to be consulted via the Internet and managed from any accredited computer. The interface provides a wealth of useful information for the scientific community about natural products, highlighting the location of species from which the compounds were isolated. Several new functionalities will be added continuously, such as new calculated molecular descriptors, and tools to aid structural elucidation using experimental and calculated NMR data and downloading several structures in one file using a batch transfer data option.

Sistemat X is freely accessible on the homepage http://sistematx.ufpb.br.

## Figures and Tables

**Table 1:** Summary of the Application Programming Interface (API) implemented by SistematX.

API	Description	Engine
**1. Structure**		
**2D drawing**	Allows drawing and visualization of chemical structures	ChemAxon
**3D generator**	Uses 2D drawing to generate a 3D representation of the molecule	ChemAxon
**3D**	Graphical visualization of 3D molecules with JavaScript	ChemDoodl
**2. Compound Identification**		
**SMILES**	Simplified Molecular Input Line Entry System	ChemAxon
**IUPAC**	IUPAC Nomenclature	ChemAxon
**InChI**	IUPAC International Chemical Identifier	ChemAxon
**InChIKey**	InChIKey is a compact format of the InChI code	ChemAxon
**CAS**	Chemical Abstracts Service Registry Number	ChemAxon
**3. Compound Data**		
**NOX**	Oxidation number (NOX) of an organic compound	ChemAxon
**Exact Mass**	Uses the mass of the most abundant isotope of each element	ChemAxon
**Relative Mass**	Uses the average atomic mass of each element	ChemAxon
**4. Geographic data**		
**Latitude**	Can be inserted by the administrator or appears by clicking in the world map	Google Inc.
**Longitude**	Can be inserted by the administrator or appears by clicking in the world map	Google Inc.
**Approximate**	Using the latitude and longitude, appears an an approximate location of the specie	Google Inc.
**Visualization**	Uses the world map to possible to visualize the localization of the species	Google Inc.
